# Gut Microbiota Changes in Metabolic Dysfunction-Associated Steatohepatitis and Inflammatory Bowel Disease: Common Pathogenic Features

**DOI:** 10.3390/cimb47100847

**Published:** 2025-10-15

**Authors:** Giuseppe Guido Maria Scarlata, Domenico Morano, Abdulrahman Ismaiel, Rocco Spagnuolo, Francesco Luzza, Dan Lucian Dumitrascu, Ludovico Abenavoli

**Affiliations:** 1 Department of Health Sciences, University of Catanzaro “Magna Graecia”, 88100 Catanzaro, Italy; giuseppeguidomaria.scarlata@unicz.it (G.G.M.S.); domenico.morano1@studenti.unicz.it (D.M.); spagnuolo@unicz.it (R.S.); luzza@unicz.it (F.L.); 2 2nd Department of Internal Medicine, “Iuliu Hatieganu” University of Medicine and Pharmacy, 400006 Cluj-Napoca, Romania; ismaiel.abdulrahman@umfcluj.ro (A.I.); ddumitrascu@umfcluj.ro (D.L.D.)

**Keywords:** gut-liver axis, microbiota, fatty liver disease, omics, pathogenesis

## Abstract

Gut microbiota changes have emerged as central players in the pathogenesis of both metabolic dysfunction-associated steatohepatitis (MASH) and inflammatory bowel disease (IBD). Although these diseases affect distinct primary organs, they share converging mechanisms driven by dysbiosis, including loss of beneficial short-chain fatty acid-producing taxa such as *Faecalibacterium prausnitzii* and *Roseburia*, enrichment of pro-inflammatory Enterobacteriaceae, and disruption of bile acid and tryptophan metabolism. These shifts compromise epithelial barrier integrity, promote the translocation of microbial products such as lipopolysaccharide, and trigger toll-like receptor 4-mediated activation of inflammatory cascades dominated by tumor necrosis factor-alpha, interleukin-6, and transforming growth factor-beta. In MASH, this dysbiotic environment fuels hepatic inflammation, insulin resistance, and fibrogenesis, while in IBD it sustains chronic mucosal immune activation. Shared features include impaired butyrate availability, altered bile acid pools affecting farnesoid X receptor and Takeda G protein-coupled Receptor 5 signaling, and defective aryl hydrocarbon receptor activation, all of which link microbial dysfunction to host metabolic and immune dysregulation. Understanding these overlapping pathways provides a deeper understanding of the role of the gut-liver and gut-immune axes as unifying frameworks in disease progression. This narrative review synthesizes current evidence on gut microbiota in MASH and IBD, underscoring the need for longitudinal, multi-omics studies and microbiome-targeted strategies to guide personalized therapeutic approaches.

## 1. Introduction

The human gut microbiota plays a critical role in maintaining intestinal homeostasis and regulating host metabolism and immune responses [1]. Disruptions in microbial composition and function, collectively referred to as gut dysbiosis, have been implicated in the development of various chronic inflammatory and metabolic disorders [2,3,4]. Among these, metabolic dysfunction-associated steatohepatitis (MASH) and inflammatory bowel disease (IBD) have gained increasing attention for their overlapping pathophysiological features linked to microbiota changes [5,6]. MASH, the progressive form of metabolic dysfunction-associated steatotic liver disease (MASLD), is characterized by hepatic steatosis, inflammation, and fibrosis, and is now one of the most common causes of chronic liver disease globally [7,8]. In this regard, MASLD is now recognized as the preferred term to describe hepatic steatosis linked to cardiometabolic abnormalities. It is diagnosed when evidence of liver fat accumulation, documented by imaging, histology, or validated biomarkers, coexists with at least one cardiometabolic risk factor such as overweight or obesity, type 2 diabetes mellitus (T2DM), hypertension, or dyslipidemia [9]. In contrast to the old non-alcoholic fatty liver disease definition, MASLD emphasizes the role of metabolic dysfunction rather than relying primarily on the exclusion of alcohol use [10]. Within the spectrum of fatty liver disease, MASH represents the progressive form, marked by steatosis along with hepatocyte ballooning and inflammation [11]. All individuals with MASH are included within the MASLD spectrum, yet only a subset of MASLD patients progress into MASH. The likelihood of progression is highest in those with cardiometabolic risk factors and genetic predispositions such as the *Patatin-like phospholipase domain-containing protein 3* I148M variant [12]. Clinically, MASLD without inflammation is often stable, while MASH carries a much higher likelihood of advancing to fibrosis, cirrhosis, and hepatocellular carcinoma (HCC) [13]. IBD, which includes Crohn’s disease (CD) and ulcerative colitis (UC), is a group of chronic immune-mediated disorders affecting the gastrointestinal tract, driven by dysregulated immune responses to intestinal microbes in genetically susceptible individuals [14]. Despite differing in clinical manifestations, both MASH and IBD exhibit characteristic gut microbiota alterations. These include reduced microbial richness and alpha diversity, depletion of beneficial anti-inflammatory taxa such as *Faecalibacterium prausnitzii* and *Akkermansia muciniphila*, and an increased abundance of pro-inflammatory pathobionts such as *Escherichia coli* (*E. coli*) and members of the Enterobacteriaceae family [15,16,17]. Functional consequences of gut dysbiosis include impaired short-chain fatty acid (SCFA) production, disrupted bile acid metabolism, and increased intestinal permeability leading to bacterial translocation and systemic inflammation [18,19,20]. Crucially, these microbial shifts influence host physiology along the gut-liver axis, modulating hepatic inflammation and metabolic homeostasis, and along the gut-immune axis as well, promoting mucosal immune dysregulation [21,22]. Key mechanisms include toll-like receptor (TLR)-mediated signaling, compromised epithelial barrier integrity, and altered levels of microbial metabolites that impact immune tone and host metabolism [23,24]. A better understanding of these shared microbial and mechanistic pathways may shed light on how microbiota-driven inflammation and metabolic dysfunction interplay in MASH and IBD. As such, the present narrative review aims to synthesize current evidence on gut dysbiosis in both conditions, highlighting common pathogenic ways.

## 2. Gut Microbiota and MASH

The disequilibrium of gut microbiota has emerged as playing a key role in the pathogenesis of MASH onset and development [6]. Indeed, MASH is associated with a distinct microbial signature, involving not only changes in taxonomic composition, but also in the functional activity of the microbiota. These changes influence hepatic inflammation, insulin resistance, and fibrogenesis through complex interactions along the gut-liver axis [25].

### 2.1. Taxonomic Shifts of Gut Microbiota in MASH

As reported in the following lines, several studies using 16S ribosomal RNA (16S rRNA) gene sequencing and metagenomic approaches have reported taxonomic alterations in MASH. Zhu et al. analyzed children with MASH, obesity, and healthy controls, finding distinct enterotypes by disease status. While healthy and obese groups differed markedly, the microbiota of obese and MASH patients overlapped. Importantly, MASH patients were enriched in Proteobacteria, Enterobacteriaceae, and *E. coli*, and uniquely showed elevated blood ethanol levels, suggesting endogenous ethanol production may contribute to liver injury [26]. Del Chierico et al. compared stool samples from obese, MASLD, MASH, and healthy children. Reduced diversity was observed in obesity and MASLD. In MASH, *Lachnospiraceae*, *Ruminococcus*, and *Dorea* were increased, while *Oscillospira* decreased in steatosis. Gas chromatography–mass spectrometry identified differences in volatile organic compounds (2-butanone, 4-methyl-2-pentanone) between MASH and controls. Microbiota-metabolite signatures distinguished healthy subjects from diseased ones, although MASLD and MASH could not be reliably separated [27,28]. Loomba et al. developed a metagenomic machine-learning model to predict MASH with fibrosis. An increase in Proteobacteria and Enterobacteriaceae, as well as a reduction in *Ruminococcus obeum* and *Eubacterium rectale*, were the strongest features. The model achieved an area under the curve (AUC) of 0.92, outperforming clinical indices [29]. In a study of biopsy-proven MASH stratified by body mass index (BMI), fibrosis ≥ F2 was linked to increased *Lactobacillus*. Compared with controls, MASH patients showed differences in *Faecalibacterium*, *Ruminococcus*, *Lactobacillus*, and *Bifidobacterium*. Lean patients had a threefold reduction in *Faecalibacterium* and *Ruminococcus*; obese patients were enriched in *Lactobacillus*; overweight patients had reduced *Bifidobacterium*. Interestingly, alpha diversity in lean MASH resembled healthy controls, pointing to distinct microbiota mechanisms in lean versus obese disease [30]. Caussy et al. combined metagenomic data from MASLD cohorts, identifying a fibrosis signature with reduced SCFA-producers (*Oscillospiraceae*, *Lachnospiraceae*, *Ruminococcus*) and enrichment of pro-inflammatory genera (*Veillonella*, *Streptococcus*, *Klebsiella*). These taxa correlated with histological severity across cohorts [31]. MASH can present in both **obese** and **lean** individuals, reflecting heterogeneity in its metabolic background. Obese MASH is typically associated with dyslipidemia, T2DM, and a stronger inflammatory and fibrotic profile, while lean MASH often occurs in individuals with normal BMI but shares metabolic risk factors such as dyslipidemia or visceral adiposity [32,33]. Relating to this, Sookoian et al. found bacterial DNA from *Roseibacillus*, *Peptostreptococcus*, *Bifidobacterium*, and *Streptomyces* enriched in obese MASLD, with the greatest increase in MASH. Proteobacteria abundance was associated with more severe disease [34]. Another study showed progressive loss of diversity from MASLD to MASH. At phylum level, both groups had more Bacteroidetes and Fusobacteria and fewer Firmicutes. At genus level, *Prevotella* decreased in MASLD, while *Megamonas* and *Fusobacterium* increased in MASH. Functional analyses revealed altered glucose metabolism and reduced flavonoid/flavonol biosynthesis in MASH [35]. Finally, in pediatric patients, MASH was linked to increased *Alistipes* and reduced Peptostreptococcaceae. Species such as *Bacteroides uniformis*, *Lachnospiraceae bacterium 7_1_58FAA*, and *Eubacterium ventriosum* were significantly higher in MASH than MASLD. The authors suggested these shifts may drive progression and could support microbiota-based diagnostic profiling [36] (Table 1).

### 2.2. Functional Alterations in Microbial Metabolism in MASH

These compositional changes suggest a loss of beneficial anti-inflammatory taxa and an enrichment of pathobionts capable of driving endotoxemia and low-grade inflammation. Taxonomic shifts in MASH are accompanied by profound functional alterations in microbial metabolism, which can be understood as being subdivided into three main areas.

1. Gut microbiota and SCFAs: one of the most consistent findings is the reduced microbial capacity to produce SCFAs, particularly butyrate, which preserves epithelial barrier integrity and exerts anti-inflammatory effects via G-protein–coupled receptor 41/43 (GPR41/43) activation and histone deacetylase (HDAC) inhibition [37,38].
2. Bile acid metabolism: MASH-associated dysbiosis is also characterized by disruption of bile acid metabolism. Microbes expressing bile salt hydrolase and 7α-dehydroxylase convert primary bile acids into secondary bile acids, such as deoxycholic acid (DCA). Elevated fecal DCA levels in MASH patients, together with a reduced abundance of *Bacteroides*, promote activation of hepatic farnesoid X receptor (FXR) and Takeda G protein–coupled receptor 5 (TGR5), enhancing pro-inflammatory and pro-fibrotic signaling [39].
3. Microbial translocation and inflammatory mediators: dysbiosis further increases intestinal permeability, as shown by downregulation of tight junction proteins (occludin, claudin-1) in both human MASH and high-fat-diet-fed mouse models. This results in higher circulating levels of lipopolysaccharide (LPS) [40]. LPS activates TLR-4 on Kupffer and hepatic stellate cells, stimulating the release of tumor necrosis factor-alpha (TNF-α), interleukin-6 (IL-6), and pro-fibrotic mediators such as transforming growth factor-beta (TGF-β), thereby linking microbial products directly to liver inflammation and fibrogenesis [41].

Overall, MASH-associated dysbiosis is defined by a concurrent reduction in microbial diversity and metabolic functionality [6]. These alterations highlight the key role of the gut microbiota in the pathogenesis of MASH and provide a rationale for the development of microbiome-targeted therapeutic strategies.

## 3. Gut Microbiota and IBD

Multiple studies employing 16S rRNA gene sequencing, shotgun metagenomics, and multi-omics approaches have consistently demonstrated that patients with IBD exhibit significant alterations in gut microbial composition when compared to healthy individuals.

### 3.1. Taxonomic Shifts of Gut Microbiota in IBD

Alterations in IBD extend beyond microbial diversity, including phylum-level shifts, expansion of pathobionts, and loss of immunomodulatory taxa. Indeed, Darfeuille-Michaud et al. first identified adherent-invasive *E. coli* (AIEC) from ileal biopsies of CD patients, showing that these strains invade epithelial cells, persist in macrophages, and trigger TNF-α, thus sustaining chronic inflammation [42]. Sokol et al. later reported a selective reduction of *F. prausnitzii* in CD, especially in post-operative recurrence, and demonstrated its ability to induce IL-10, supporting an anti-inflammatory role [43]. In treatment-naïve pediatric CD, Gevers et al. showed that biopsy microbiota better detected disease than stool, with enrichment of Enterobacteriaceae, *Fusobacterium*, and *Peptostreptococcus*, and depletion of *Bacteroides* and *Faecalibacterium* [44]. Machiels et al. found UC patients had reduced butyrate producers (*Roseburia hominis*, *F. prausnitzii*) and increased mucin-degraders (*Ruminococcus gnavus*) [45]. In mice, germ-free conditions prevented ileitis, antibiotics reduced it, and microbiota transfer from diseased donors induced it. Gut dysbiosis was associated with loss of Paneth cell antimicrobial function, while *E. coli* LF82 alone was insufficient, highlighting the causal role of complex microbial communities [46]. Forbes et al. confirmed reduced *Clostridia* and *Bacteroides* with increased Enterobacteriaceae across immune-mediated disorders but also identified IBD-specific signatures [47]. Data from the IBD Multi-omics Database showed depletion of Firmicutes (*F. prausnitzii*, *Roseburia*, *Eubacterium hallii*) and expansion of Proteobacteria (*E. coli*, *K. pneumoniae*), correlating with disease activity and flares [48]. A meta-analysis of ~2000 metagenomes supported disease-specific functional profiles across gut–liver axis disorders. Patients showed reduced pathways for SCFA and bile acid metabolism and enrichment of genes related to oxidative stress and pro-inflammatory metabolites. While some alterations overlapped with other gut-liver disorders, IBD had a distinct loss of symbiotic taxa and expansion of pathobionts, reinforcing the role of the microflora in intestinal inflammation [49]. Network-based analyses further showed reduced Firmicutes, Bacteroidetes, and Actinobacteria in IBD, with depleted taxa highly connected in microbial networks, suggesting that their loss destabilizes community structure [50]. In remission, IBD patients still showed lower alpha diversity, distinct beta diversity, and enrichment of flavonoid-degraders, while healthy controls had more *Akkermansia*, *Oscillibacter*, and *Coprococcus*. Enterobacteriaceae remained central in microbial networks, supporting persistent low-grade inflammation [51]. A recent study confirmed reduced richness and depletion of *Subdoligranulum*, *Ruminococcus*, *Anaerostipes*, and *Lachnospira*. UC patients showed more *Streptococcus* but fewer *Alistipes*, while CD patients were enriched in *Lachnoclostridium*, *Fusobacterium*, *Cloacibacillus*, and *Erysipelatoclostridium*, with reduced *Faecalibacterium*, *Roseburia*, and *Haemophilus*. Active disease showed further depletion of anti-inflammatory taxa (*Roseburia*, *Coprococcus*, *Ruminiclostridium*) [52]. Moreover, extraintestinal manifestations were linked to decreased *Agathobacter* and *Blautia* and increased *Eggerthella* [53]. Finally, Italian data showed an increased Firmicutes/Bacteroidetes (F/B) ratio in IBD, suggesting its potential role as a biomarker [54,55] (Table 2).

### 3.2. Functional Alterations in Microbial Metabolism in IBD

Beyond compositional changes, IBD-associated dysbiosis is also characterized by functional disruptions in microbial metabolism, which can be described as being divided into four main areas.

1. Gut microbiota and SCFAs: metagenomic and metabolomic analyses consistently demonstrate reduced production of SCFAs, particularly butyrate and propionate [56,57]. Butyrate, mainly produced by *F. prausnitzii*, *Roseburia*, and *Eubacterium rectale*, serves as the primary energy source for colonocytes and exerts potent anti-inflammatory effects. Its depletion impairs epithelial barrier integrity and exacerbates mucosal inflammation in IBD [58].
2. Redox imbalance: IBD microbiota displays increased oxidative stress and preferential use of nitrate and sulfate respiration, creating conditions that favor facultative anaerobes such as Enterobacteriaceae over obligate anaerobes. This shift in redox balance reinforces microbial instability and drives chronic immune activation [59].
3. Bile acid metabolism: in healthy individuals, microbial deconjugation and 7α-dehydroxylation convert primary bile acids into secondary ones such as DCA and lithocholic acid. In IBD, these pathways are impaired, resulting in an accumulation of conjugated bile acids that promote barrier dysfunction and mucosal inflammation through FXR and TGR5 signaling [60].
4. Tryptophan metabolism and immune regulation: microbial catabolism of tryptophan into indole derivatives is a major source of ligands for the aryl hydrocarbon receptor (AhR), which maintains intestinal immune tolerance and epithelial repair. Loss of this pathway in IBD reduces AhR activation, contributing to chronic inflammation [61]. In addition, multi-omics evaluations reveal that strain-level variation modulates functional outputs. For example, *Ruminococcus gnavus*, commonly enriched in IBD, may exert either pro- or anti-inflammatory effects depending on capsular polysaccharide expression [62].

These findings suggest that microbial function, and not only taxonomy, plays a pivotal role in modulating host inflammation and disease onset.

## 4. Shared Pathogenic Mechanisms of Gut Dysbiosis in MASH and IBD

**Figure 1:**
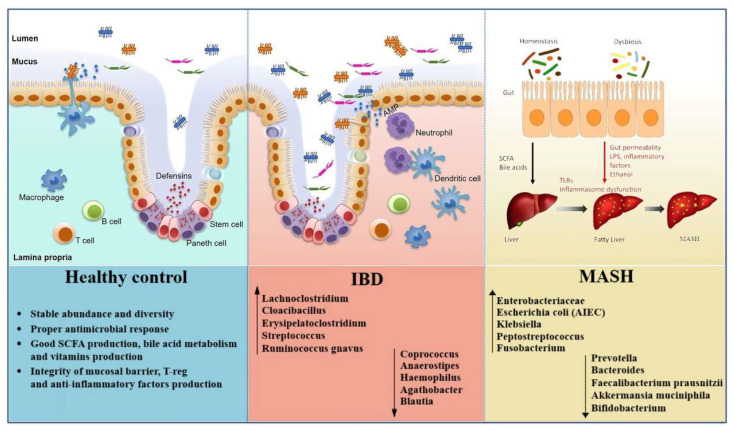
Alterations of gut microbiota and mucosal barrier integrity in health, IBD, and MASH. Healthy controls show stable microbial diversity, SCFA production, and intact mucosal defenses. IBD is characterized by gut dysbiosis with expansion of pro-inflammatory taxa and loss of beneficial microbes, leading to barrier disruption and immune activation. In MASH, enrichment of pathobionts and depletion of protective species promote gut permeability, endotoxemia, and inflammasome dysfunction, driving liver steatosis and progression to MASH.

Although MASH and IBD affect distinct primary organs, converging evidence indicates that they share a core pattern of gut dysbiosis that disrupts epithelial integrity, modulates innate and adaptive immune responses, and alters host metabolism along both the gut-liver and gut-immune axes, as reported in Figure 1 [1,3,63].

Multi-omics studies in both conditions have consistently demonstrated a loss of beneficial commensals, particularly butyrate-producing taxa such as *F. prausnitzii*, *Roseburia* spp., and *Eubacterium rectale*, as well as mucin-degrading yet homeostasis-promoting species like *Akkermansia muciniphila*, together with an expansion of pro-inflammatory Proteobacteria (e.g., *E. coli*, *Klebsiella* spp.) and other members of the *Enterobacteriaceae* family [3,4,57]. In this regard, experimental analyses demonstrate their causal role in driving chronic intestinal and hepatic inflammation via barrier disruption, metabolic signaling interference, and immune activation [58,59,60]. One of the most consistent findings across MASH and IBD is the depletion of SCFA bacterial producers, with reductions in butyrate and propionate impairing colonocyte energy metabolism, HDAC inhibition, and GPR41/43 signaling, thereby weakening tight-junctions, thinning the mucus layer, and reducing regulatory T-cell differentiation and IL-10 production [57,58,64]. The consequent barrier breach facilitates microbial translocation, allowing bacterial components such as LPS to reach the portal and systemic circulation, amplifying hepatic inflammation in MASH and mucosal inflammation in IBD [41,60,65]. Gut dysbiosis in both conditions is also associated with reduced bile salt hydrolase and 7α-dehydroxylase activities, leading to impaired generation of secondary bile acids and altered bile acid receptor signaling via FXR and TGR5 [39,60,66]. These changes drive pro-inflammatory cytokine release and hepatic stellate cell activation in MASH, while exacerbating epithelial barrier dysfunction and mucosal immune activation in IBD [67]. In parallel, the disruption of intestinal barrier integrity permits microbe-associated molecular patterns (MAMPs), particularly LPS from Gram-negative bacteria, to engage TLR4 on Kupffer cells and hepatic stellate cells in MASH, as well as on lamina propria myeloid cells in IBD. This interaction drives NF-κB activation and perpetuates an inflammatory cascade dominated by TNF-α, IL-6, and TGF-β [59,67]. Inflammation-driven redox shifts in both diseases lead to the accumulation of host-derived electron acceptors such as nitrate and tetrathionate, favoring the expansion of facultative anaerobes, particularly *Enterobacteriaceae* [41]. In CD, AIEC exploits this niche to persist intracellularly, while in MASH, ethanol-producing *E. coli* can potentiate oxidative stress and hepatocellular injury, reinforcing a Proteobacteria-rich, pro-inflammatory state [24,51]. Another shared mechanism involves disruption of the AhR axis: microbial catabolism of tryptophan into indole derivatives produces ligands that promote epithelial regeneration, mucus secretion, and IL-22-mediated immune tolerance [61,68]. Reduced microbial indole production and impaired AhR signaling are well established in IBD, and emerging evidence indicates similar deficits in MASH, with decreased indolepropionic acid correlating with fibrosis progression [68,69]. Comparative microbiota evaluations in patients with both IBD and fatty liver disease reveal overlapping signatures, including depletion of *Subdoligranulum*, *Parabacteroides*, and *Lachnospira*, and enrichment of *Alistipes*, *Odoribacter*, and *Sutterella* [54]. These findings suggest bidirectional modulation, whereby chronic gut inflammation exacerbates hepatic dysbiosis and metabolic stress, while metabolic dysfunction further compromises mucosal immune regulation in IBD [54,70]. At the same time, it should be noted that the gut microbiota undergoes changes from the stage of MASLD up to the development of HCC, as reported in Table 3 [71,72,73,74].

**Figure 2:**
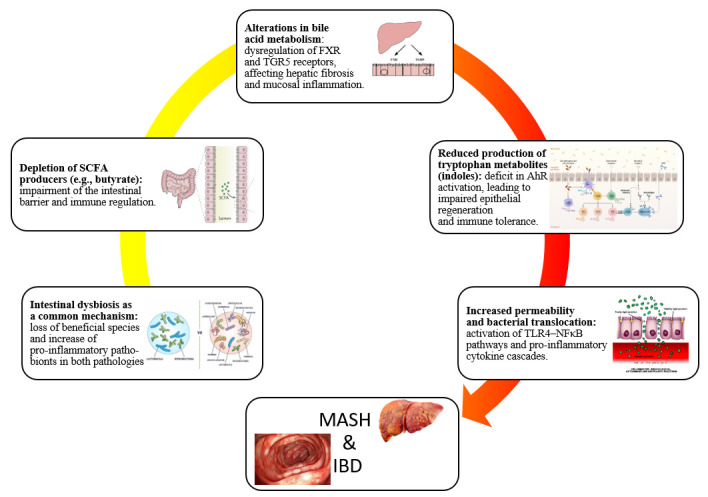
Schematic representation of the main shared pathways in IBD-MASH.

Despite these insights, systematic, longitudinal multi-omics analyses of IBD–MASH coexistence remain scarce, constituting a critical research gap. Overall, MASH and IBD converge on a shared dysbiotic framework characterized by (i) depletion of SCFA-producing taxa with consequent loss of epithelial barrier integrity and immunoregulatory support, (ii) dysregulation of bile-acid pools and receptor signaling via FXR and TGR5, (iii) barrier breakdown and systemic translocation of MAMPs leading to LPS-TLR4 activation, (iv) inflammation-driven redox shifts favoring pathogenic Enterobacteriaceae expansion, and (v) disruption of the tryptophan-AhR metabolic axis with impaired IL-22–mediated mucosal protection (Figure 2).

## 5. Conclusions and Future Perspectives

To the best of our knowledge, this narrative review is the first to specifically address the changes in gut microbiota in the context of both MASH and IBD. While previous reviews have examined these conditions separately, our work uniquely integrates them within a shared pathogenic framework, emphasizing overlapping microbial, metabolic, and immune pathways. As such, the present review could prove interesting to both researchers and clinicians. The complex interplay between gut dysbiosis, intestinal barrier dysfunction, and immune-metabolic signaling emerges as a shared pathogenic framework linking MASH and IBD. Despite differences in their primary target organs, both conditions converge on common microbial, metabolic, and inflammatory pathways that perpetuate chronic tissue injury. Advances in multi-omics technologies have deepened our understanding of these shared mechanisms, yet translational integration into clinical practice remains limited. Future research should prioritize large-scale, longitudinal studies in diverse populations to dissect the temporal sequence of dysbiosis, barrier disruption, and host response in MASH–IBD coexistence. In this regard, comparative studies evaluating microbiota-targeted therapies, ranging from dietary interventions, prebiotics to live biotherapeutics, bile acid modulators and fecal microbiota transplantation, are warranted to define personalized treatment strategies [75,76,77]. Furthermore, leveraging machine learning-driven microbiome profiling may enable early disease detection, risk stratification, and therapy optimization [78,79]. Closing these knowledge gaps will play an essential role in translating mechanistic insights into effective interventions, ultimately improving outcomes for patients affected by these interconnected gut–liver–immune disorders.

## Figures and Tables

**Table 1:** Summary of the studies about the gut microbiota composition in MASH.

Model	Study Groups	Main Findings	Reference
Human	MASH vs. obese vs. healthy controls	Distinct enterotypes based on disease status; MASH enriched in Proteobacteria, Enterobacteriaceae, *E. coli*; elevated blood ethanol only in MASH group, suggesting endogenous ethanol production as a pathogenic mechanism; overlap in microbial signatures supports a continuum from obesity to MASH.	[26]
Human	Obese vs. MASLD vs. MASH vs. healthy controls	Reduced microbial diversity in obesity and MASLD; MASH enriched in Lachnospiraceae, *Ruminococcus*, *Dorea*; decreased *Oscillospira* in steatosis; volatile organic compounds (2-butanone, 4-methyl-2-pentanone) differed between MASH and controls; microbiota/metabolite signatures distinguished healthy vs. diseased, but prediction model couldn’t distinguish MASLD from MASH.	[27]
Human	MASLD patients with/without MASH-fibrosis	Increased Proteobacteria, Enterobacteriaceae; decreased *Ruminococcus obeum*, *Eubacterium rectale* in MASH-fibrosis group; machine-learning prediction model achieved AUC = 0.92, outperforming standard clinical scores.	[29]
Human	Biopsy-proven MASH patients stratified by BMI	Fibrosis ≥ F2 linked to *Lactobacillus* increase; lean MASH: ↓ *Faecalibacterium*, ↓ *Ruminococcus*, ↓ *Lactobacillus*; obese MASH: ↑ *Lactobacillus*; overweight MASH: ↓ *Bifidobacterium*; lean MASH patients showed alpha diversity comparable to healthy controls, suggesting distinct microbiota-related mechanisms in lean vs. obese phenotypes.	[30]
Human	MASLD and MASH cohorts	Advanced fibrosis linked to ↓ SCFA-producing taxa (*Oscillospiraceae*, *Lachnospiraceae*, *Ruminococcus*) and ↑ pro-inflammatory genera (*Veillonella*, *Streptococcus*, *Klebsiella*); microbial taxa consistently associated with histological severity across cohorts.	[31]
Human	Obese MASLD and MASH groups	↑ *Roseibacillus*, *Peptostreptococcus*, *Bifidobacterium*, *Streptomyces* in MASH; Proteobacteria linked to severe phenotype; obese MASH associated with stronger inflammatory/fibrotic profile compared to lean MASH.	[34]
Human	MASLD vs. MASH	Both MASLD and MASH: ↑ Bacteroidetes, ↑ Fusobacteria, ↓ Firmicutes; MASH: ↑ *Megamonas*, ↑ *Fusobacterium*; MASLD: ↓ *Prevotella*; MASH also showed altered glucose metabolism pathways and impaired flavonoid/flavonol biosynthesis, indicating functional as well as taxonomic dysbiosis.	[35]
Human	Pediatric MASLD vs. pediatric MASH	MASH: ↑ *Alistipes*, ↓ Peptostreptococcaceae; ↑ *Bacteroides uniformis*, ↑ *Lachnospiraceae bacterium 7_1_58FAA*, ↑ *Eubacterium ventriosum*, ↑ *Roseburia*; findings suggest broader microbial shifts may be crucial in progression and improvement of MASLD/MASH, supporting microbiota-based diagnostic profiling.	[36]

**Abbreviations:** AUC, area under the curve; *E. coli*, *Escherichia coli*; MASLD, metabolic dysfunction-associated steatotic liver disease; MASH, metabolic dysfunction-associated steatohepatitis; BMI, body mass index; SCFA, short-chain fatty acid; up arrow, increased abundance or levels; down arrow, decreased abundance or levels.

**Table 2:** Summary of the studies about the gut microbiota composition in IBD.

Model	Study Groups	Main Findings	Reference
Human	CD patients	Isolation of AIEC from ileal biopsies; AIEC adhere to and invade epithelial cells, survive in macrophages, induce TNF-α and chronic inflammation; AIEC strains implicated in initiating and perpetuating ileal inflammation.	[42]
Human	IBD patients vs. healthy controls	Reduced *F. prausnitzii* abundance in CD group, especially in post-operative recurrence; *F. prausnitzii* induces IL-10 with anti-inflammatory action; selective reduction suggests loss of key butyrate-producer contributes to recurrence risk.	[43]
Human	Pediatric treatment-naïve CD vs. controls	Ileal mucosa enriched in Enterobacteriaceae, *Fusobacterium*, *Peptostreptococcus*; depleted *Bacteroides*, *Faecalibacterium* in CD group; biopsy-associated microbiota distinguished CD more effectively than fecal samples.	[44]
Human	UC patients vs. healthy controls	↓ Butyrate-producers (*Roseburia hominis*, *F. prausnitzii*); ↑ Mucin-degraders (*Ruminococcus gnavus*); microbial imbalance reflects impaired SCFA production and mucosal barrier degradation in UC.	[45]
Animal	Mice models of CD-like ileitis	Germ-free mice protected; antibiotics reduce ileitis; transfer of dysbiotic microbiota induces ileitis; *E. coli* LF82 on its own is insufficient; inflammation requires complex microbial communities; gut dysbiosis downregulates Paneth cell antimicrobial genes (lysozyme, cryptdin-2).	[46]
Human	IBD vs. other immune-mediated inflammatory disorders	↓ *Clostridia*, ↓ *Bacteroides*; ↑ Enterobacteriaceae; core shifts shared with other immune disorders but also IBD-specific alterations, indicating both shared and disease-specific dysbiosis.	[47]
Human	IBD patients vs. controls	↓ Firmicutes (*F. prausnitzii*, *Roseburia*, *Eubacterium hallii*); ↑ Proteobacteria (*E. coli*, *Klebsiella pneumoniae*); compositional changes correlated with disease activity, more pronounced during flares.	[48]
Human	IBD patients vs. controls	Altered composition across Firmicutes, Bacteroidetes, Proteobacteria, Actinobacteria; families enriched in IBD showed poor network connectivity, whereas depleted taxa were highly interconnected, supporting loss of ecosystem stability.	[50]
Human	IBD in clinical remission vs. healthy controls	↓ Alpha diversity, distinct beta diversity; healthy: ↑ *Akkermansia*, *Oscillibacter*, *Coprococcus*; IBD: ↑ Flavonoid-degraders; Enterobacteriaceae formed highly interconnected modules sustaining subclinical inflammation even in remission.	[51]
Human	UC vs. CD	UC: ↑ *Streptococcus*, ↓ *Alistipes*; CD: ↑ *Lachnoclostridium*, *Fusobacterium*, *Cloacibacillus*, *Erysipelatoclostridium*; ↓ *Faecalibacterium*, *Roseburia*, *Haemophilus*; activity-dependent: further ↓ anti-inflammatory taxa (*Roseburia*, *Coprococcus*, *Ruminiclostridium*) in active disease vs. remission.	[52]
Human	IBD with extraintestinal manifestations	↓ *Agathobacter*, ↓ *Blautia*; ↑ *Eggerthella*; specific dysbiosis pattern associated with extraintestinal manifestations.	[53]
Human	IBD vs. healthy controls	↑ F/B ratio; F/B imbalance suggested as potential biomarker for IBD.	[54]

**Abbreviations:** AIEC, adherent-invasive *Escherichia coli*; CD, Crohn’s disease; F/B ratio, Bacteroidetes/Firmicutes ratio; *F. prausnitzii*, *Faecalibacterium prausnitzii*; IBD, inflammatory bowel disease; IL-10, interleukin-10; TNF-α, tumor necrosis factor-alpha; UC, ulcerative colitis; up arrow, increased abundance or levels; down arrow, decreased abundance or levels.

**Table 3:** Summary of the gut microbiota changes at different stages of liver disease.

Disease Stage	Gut Microbiota Changes	Reference
MASLD	Increased abundance of Proteobacteria and Firmicutes; decreased Bacteroidetes; enrichment of *Escherichia* and *Klebsiella* spp.; reduced microbial diversity; decline of SCFA-producing genera (*Roseburia*, *Faecalibacterium*); functional signatures include impaired bile acid metabolism and enhanced LPS-mediated endotoxemia.	[71]
MASH	Increase of Proteobacteria; reduction of Bifidobacterium and *F. prausnitzii*; enrichment of pro-inflammatory taxa (*Dorea*, *Ruminococcus*, *Megamonas*); decreased abundance of Oscillospiraceae; functional alterations include reduced butyrate and propionate biosynthesis, impaired flavonoid metabolism, and increased ethanol production.	[72]
Liver fibrosis	Enrichment of *Streptococcus* and *Veillonella* spp.; reduction in butyrate-producing species (*Roseburia*, *Eubacterium rectale*); increased ethanol-producing bacteria (*Klebsiella pneumoniae*); reduced microbial diversity and expansion of lactic acid-producing bacteria.	[71]
Liver cirrhosis	Dominance of Enterobacteriaceae and Streptococcaceae; depletion of autochthonous taxa (Lachnospiraceae, Ruminococcaceae); increased oral-origin taxa (*Veillonella*, *Streptococcus*, *Prevotella*); reduced *Akkermansia muciniphila*; decreased SCFA production and increased endotoxin release.	[73]
HCC	Increased abundance of *E. coli*, *Enterococcus faecalis*, and *Klebsiella* spp.; lower levels of beneficial SCFA-producing bacteria (*Faecalibacterium*, *Roseburia*, *Blautia*); enrichment of pro-inflammatory and carcinogenesis-associated taxa (*Bacteroides*, *Fusobacterium*, *Clostridium* cluster XI); microbiota signatures linked to secondary bile acid overproduction, DNA-damaging metabolites, and impaired immune surveillance.	[74]

**Abbreviations:** MASLD, Metabolic dysfunction-Associated Steatotic Liver Disease; MASH, Metabolic dysfunction-Associated Steatohepatitis; *F. prausnitzii*, *Faecalibacterium prausnitzii*; HCC, Hepatocellular Carcinoma; SCFA, Short-Chain Fatty Acid; LPS, Lipopolysaccharide.

## Data Availability

No new data were created or analyzed in this study. Data sharing is not applicable to this article.

## Abbreviations

The following abbreviations are used in this manuscript:

16S rRNA	16S ribosomal RNA
AhR	Aryl hydrocarbon Receptor
AUC	Area Under the Curve
BMI	Body mass index
CD	Crohn’s disease
DCA	Deoxycholic Acid
*E. coli*	*Escherichia coli*
F/B	Firmicutes/Bacteroidetes ratio
FXR	Farnesoid X Receptor
GPR41/43	G-protein–coupled Receptor 41/43
HCC	Hepatocellular carcinoma
HDAC	Histone Deacetylase
IBD	Inflammatory Bowel Disease
IL	Interleukin
LPS	Lipopolysaccharide
MAMPs	Microbe-Associated Molecular Patterns
MASH	Metabolic dysfunction-Associated Steatohepatitis
MASLD	Metabolic dysfunction-Associated Steatotic Liver Disease
SCFA	Short-Chain Fatty Acid
T2DM	Type 2 diabetes mellitus
TGF-β	Transforming Growth Factor-beta
TGR5	Takeda G protein-coupled Receptor 5
TLR	Toll-Like Receptor
TNF-α	Tumor Necrosis Factor-alpha
UC	Ulcerative Colitis
